# rLj-RGD3, a Novel Three-RGD-Motif-Containing Recombinant Protein from* Lampetra japonica*, Protects PC12 Cells from Injury Induced by Oxygen-Glucose Deprivation and Reperfusion

**DOI:** 10.1155/2016/6701249

**Published:** 2016-09-05

**Authors:** Li Lv, Qian Lu, Fangyu Shao, Weiping Li, Qin Zhou, Jihong Wang, Qingwei Li

**Affiliations:** ^1^School of Life Sciences, Liaoning Normal University, Dalian, Liaoning 116029, China; ^2^Department of Pharmacology, Dalian Medical University, Dalian, Liaoning 116044, China; ^3^College of Basic Medicine, Jilin Medical University, Jilin, Jilin 132013, China

## Abstract

rLj-RGD3 is a 14.5 kDa recombinant protein with 3 RGD (Arg-Gly-Asp) motifs from the salivary gland secretions of* Lampetra japonica*, which is a histidine-rich and arginine-rich protein. Previous reports indicated that rLj-RGD3 has typical functions of RGD-toxin protein, such as platelet aggregation suppression tumour metastasis and angiogenesis inhibition. Because histidine and arginine have cerebral ischemia-reperfusion and neuroprotective functions, we investigated whether rLj-RGD3 has such activities and studied the mechanism. The effects of rLj-RGD3 on neuroprotection and antiapoptosis were determined. The expression level of focal adhesion kinase (FAK), p-FAK, Caspase-3, and Bcl-2 after oxygen-glucose deprivation and reperfusion (OGD-R) was examined. The viability of PC12 cells incubated with rLj-RGD3 at high concentrations (16 *μ*mol/L) increased significantly due to its ability to protect the cells from apoptosis after OGD-R-induced injury. Furthermore, rLj-RGD3 attenuated the damage due to OGD-R. Most of the PC12 cells were apoptotic after OGD-R. In contrast, the number of apoptotic PC12 cells was significantly decreased in the group treated with a high-dose of rLj-RGD3. In addition, rLj-RGD3 activated FAK and p-FAK protein. rLj-RGD3 inhibited Caspase-3 and upregulated Bcl-2 protein expression in PC12 cells after OGD-R. The study provides the first evidence for neuroprotective effects of rLj-RGD3 in ischemic injury that may be partly mediated through inhibition of Caspase-3 and upregulation of Bcl-2, FAK, and p-FAK protein expression.

## 1. Introduction

Ischemic stroke, commonly known as cerebral infarction, is a cerebral insufficiency caused by the brain tissue ischemia and hypoxia associated with the clinical syndrome. The main clinical manifestations include sensory loss, facial paralysis, ataxia, swallowing difficulties, speech problems, and visual disorders. Ischemic stroke has become the third largest cause of death in humans [1], and its incidence continues to increase yearly. Therefore, the development of drugs that provide effective protection from hypoxic-ischemic brain damage and inhibition of neuronal apoptosis is needed.

The integrin family of cell adhesion molecules primarily mediates the adhesion between cells and between the cells and the extracellular matrix (ECM), as well as inducing bidirectional signalling between the ECM and cells [2]. Integrin-mediated adhesion regulates many cell functions such as apoptosis [3], proliferation, adhesion, and migration [4], including lymphocyte homing. Currently, specific binding sites between extracellular matrix proteins and the integrin receptors have been identified, namely, the arginine-glycine-aspartic sequence (Arg-Gly-Asp, RGD). A polypeptide containing the RGD sequence can recognise the integrin receptor and bind to it, thereby influencing cellular biological functions [5].

An increasing number of investigations have been committed to the development of RGD peptide-containing drugs. In recent years, a large number of studies have shown that RGD peptides can inhibit tumour cell metastasis, vascular endothelial cell growth, osteoclast activity, and platelet aggregation [6, 7].

rLj-RGD3 is a 14.5 kDa recombinant protein with 3 RGD (Arg-Gly-Asp) motifs from the salivary gland secretions of* Lampetra japonica*, which is a histidine-rich and arginine-rich protein. Previous study results investigated that rLj-RGD3 has typical functions of RGD-toxin protein, such as platelet aggregation suppression, tumour metastasis, and angiogenesis inhibition [8]. Because histidine and arginine have cerebral ischemia-reperfusion and neuroprotective functions, we investigated whether rLj-RGD3 has such activities on the focal cerebral ischemia and studied the mechanism, such as the role of FAK in the regulation of the integrin-PI3K/Akt pathway by rLj-RGD3 in the OGD-R-induced PC12 cells damage model.

## 2. Materials and Methods

### 2.1. Materials

The recombinant plasmids pET23b-RGD3 are preserved in our laboratory. Histidine affinity chromatography columns (His. Bind Column) were purchased from Novagen, Tricine, and isopropyl *β*-D-1-thiogalactopyranoside (IPTG) were purchased from Amresco.

### 2.2. Extraction and Purification of the rLj-RGD3 Proteins

BL21* E. coli* cells containing the recombinant plasmids pET23b-RGD3 were cultured until the cells reached the logarithmic phase of growth, which corresponded to an OD600 value (optical density value at 600 nm) of 0.6. Subsequently, isopropyl *β*-D-1-thiogalactopyranoside (IPTG) was added to a final concentration of 1 mM, and the cells were cultured at 30°C overnight to induce the expression of soluble rLj-RGD3. The BL21 cells were then sonicated and centrifuged, and the target proteins were purified via histidine affinity chromatography according to the manufacture's protocol.

The purified rLj-RGD3 were identified through Tricine-SDS PAGE in the presence of glycerol, which is commonly used to separate proteins with low molecular weights. The experimental procedure included gel preparation, sample loading, fixation, staining, and destaining until clear protein bands were visible.

### 2.3. Cell Culture

Normal PC12 cells were purchased from the Institute of Cell Biology, Chinese Academy of Sciences (Shanghai), and maintained in Dulbecco's Modified Eagle's Medium (DMEM, Sigma, USA) supplemented with 5% heat-inactivated foetal calf serum (FCS, Sigma, USA), 10% heat-inactivated horse serum, penicillin (50 U/mL), and streptomycin (50 mg/L). PC12 cells were cultured at 37°C in a humidified atmosphere of 5% CO_2_ and 95% air. The cells were divided into six groups: the control group; the OGD-R model group; the rLj-RGD3 high-dose (16 *μ*mol/L), medium-dose (8 *μ*mol/L), and low-dose (4 *μ*mol/L) groups; and the edaravone (23 *μ*mol/L) positive control group.

### 2.4. Oxygen-Glucose Deprivation and Reperfusion

PC12 cells were subjected to OGD-R as described previously [9]. Briefly, the original medium was removed, and the cells were washed with Earle's balanced salt solution (EBSS) at pH 7.4 and placed in fresh glucose-free EBSS supplemented with Na_2_S_2_O_4_. The culture dishes were then introduced in a mixture of 5% CO_2_ and 95% air at 37°C for 2 h, after which the medium was replaced with fresh DMEM for another 24 h. The control culture was maintained in normal EBSS and incubated under the normal conditions. Edaravone was added to the culture 30 min before the OGD treatment.

### 2.5. Cell Viability

Cellular viability was evaluated using 2-(4,5-dimethylthiazol-2-yl)-2,5-diphenyltetrazolium bromide (MTT; Sigma Corp., USA). The MTT reagent was dissolved in phosphate-buffered saline (PBS) and added to the culture at a final concentration of 0.5 mg/mL. After an additional 4 h incubation at 37°C, the media were carefully removed, and the triple fluid (10 g sodium dodecyl sulphonate, 5 mL of isobutanol, and a drop of hydrochloric acid, volume adjusted to 100 mL) was added to each well. The OD was measured on a plate reader at 570 nm after 15 h. The results are expressed as the percentages of the control.

### 2.6. LDH Activation Assays

Damage to the cells results in the release of lactate dehydrogenase (LDH). Therefore, the LDH activity in the supernatant fluid rises significantly. The total LDH activity in the supernatants was measured using the LDH Activity Assay Kit (Nanjing Jiancheng Corp., China) in accordance with the manufacturer's instructions. The results are expressed as U/g protein.

### 2.7. Determination of Reduced GSH

GSH is an important free radical scavenger and antioxidant in vivo. It has antioxidant, antiaging, immunity-enhancing, and other physiological functions. The cells (1 × 10^5^/mL) were harvested after treatment with OGD for 2 h and reperfusion for 24 h. The cells were washed twice using cold PBS. The concentration of GSH in the cells was determined using a commercial assay kit (Nanjing Jiancheng Corp., China) according to the manufacturer's instructions and expressed as g of GSH/L.

### 2.8. Acridine Orange/Ethidium Bromide (AO/EB) Staining

Sterilised coverslips were placed into 6-well plates, which were then placed in a laminar flow UV disinfection system overnight. Normal PC12 cells were plated into 6-well plates at a density of 1 × 10^5^/mL cell. The model was performed as described above.

Acridine orange (AO) can penetrate the cell membrane and bind to DNA, with the result that the cells appear green. In contrast, ethidium bromide (EB) only can access DNA through a damaged cell membrane, labelling the cells with an orange-red colour that is brighter than the green AO stain. AO and EB were each dissolved in 10 mL of PBS at a concentration of 10 mg/mL. Equal volumes of the two solutions were mixed together before use. After a 24 h OGD-R incubation, the coverslips were gently removed from the wells with tweezers, and 10 *μ*L of the AO/EB mixture was applied. Then, the coverslip was inverted onto a glass slide. The images were acquired using fluorescence microscopy.

### 2.9. Flow Cytometric Analysis

For the quantitative assessment of apoptosis, Annexin V-FITC and PI double-staining by flow cytometry were performed. Briefly, 5 × 10^6^/mL PC12 cells were washed three times with cold PBS (4°C) and resuspended in 300 *μ*L of binding buffer (BB). The cells were labelled according to the kit instructions (Beyotime Bio Co., Beijing, China). The cells were analysed immediately using flow cytometry. The signals from apoptotic cells are localised in the lower right quadrant of the resulting dot-plot graph [10].

### 2.10. Western Blot Analysis

Equal amounts of protein (60 *μ*g) were separated on 12% SDS-PAGE gels for detection of FAK and p-FAK and 15% gels for Caspase-3 and Bcl-2 protein levels and then transferred to nitrocellulose membranes. The membranes were then blocked with 5% skim milk in PBS containing 0.1% Tween 20 (PBS-T). After washing with PBS-T, the membranes were probed overnight at 4°C with primary antibodies against FAK, p-FAK, Caspase-3, or Bcl-2 (all at a 1 : 1000 dilution). GAPDH was also detected with appropriate primary antibodies to ensure equal loading of cytoplasmic and nuclear proteins. Horseradish peroxidase-conjugated secondary antibodies (all at a 1 : 2000 dilution) were then applied to membranes, and the blots were developed with DAB. The expression level of each protein was determined by analysing the signal captured on the membrane using a gel imaging system.

### 2.11. Statistical Analyses

All values are expressed as the mean ± SEM. Statistical comparisons were made using a one-way ANOVA test, followed by the Student-Newman-Keuls (SNK) test. In all cases, a difference was considered significant when *p* was less than 0.05. All statistical analyses were conducted using the SPSS16 statistical software package (SPSS, Inc., Chicago, IL, USA).

## 3. Results

### 3.1. Expression and Purification of rLj-RGD3

**Figure 1:**
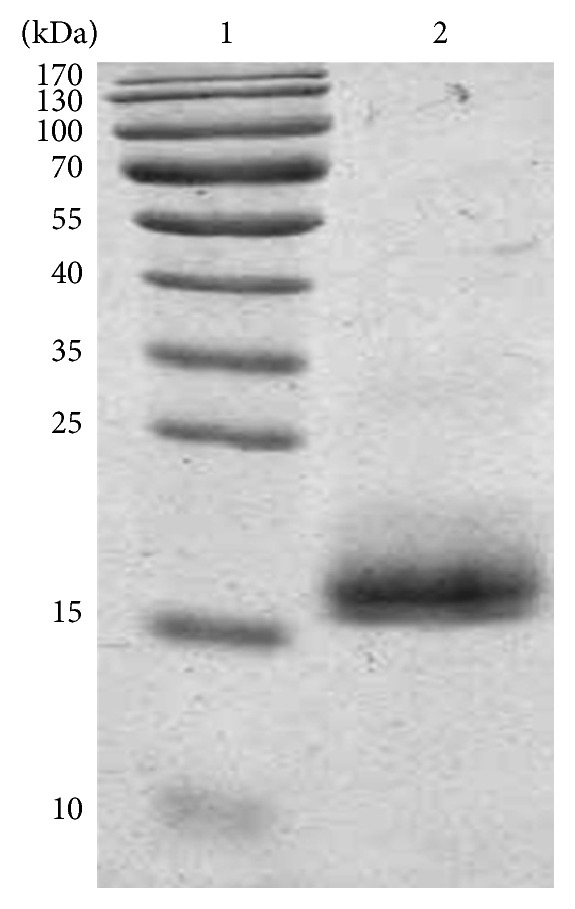
Tricine SDS-PAGE result of purified rLj-RGD3. Lane 1: protein marker; Lane 2: 14.5 kDa of purified rLj-RGD3.

rLj-RGD3 was expressed as a soluble fusion protein in* E. coli* BL21 cells and purified via histidine affinity chromatography. The sequence of purified rLj-RGD3 was confirmed by NH2-terminal amino acid sequencing. The protein was approximately 14.5 kDa and migrated as a single band on a Tricine SDS-PAGE gel (Figure 1). The concentration of purified rLj-RGD3 was 15 *μ*g/*μ*L.

**Figure 2:**
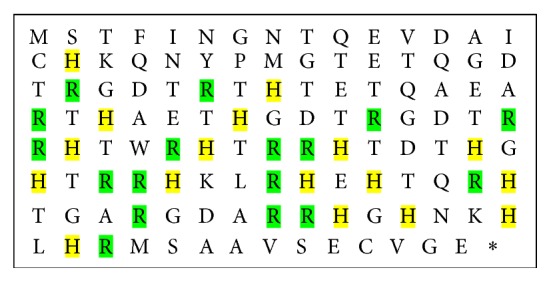
The amino acid sequence of rLj-RGD3. Histidines are highlighted in yellow; arginines are highlighted in green.

The amino acid sequence of Lj-RGD3 contains 118 amino acids including 2 cysteines, 17 histidine, 17 arginine, and 20 threonine as well as 3 RGD motifs (Figure 2). The histidine-rich and arginine-rich characteristics of Lj-RGD3 suggested that it will have the functions of cerebral ischemia-reperfusion and neuroprotection.

### 3.2. Effect of rLj-RGD3 on PC12 Cell Viability after OGD-R

**Figure 3:**
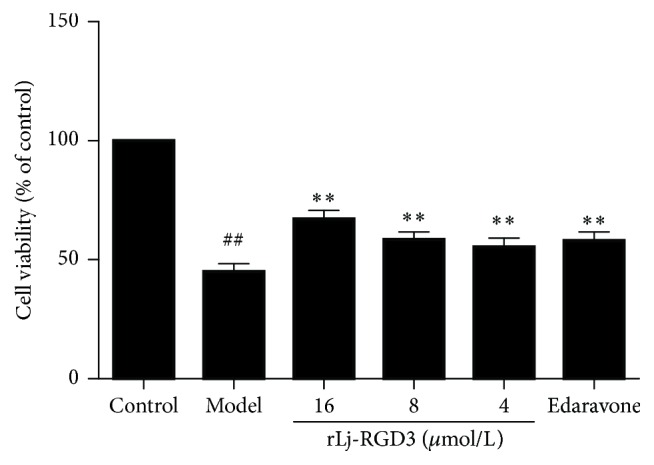
The effect of rLj-RGD3 on PC12 cell viability after OGD-R-induced injury ($\bar{X}\pm SD$, *n* = 6). Normal PC12 cells were subjected to OGD for 2 h and reperfusion for 24 h. PC12 cell viability was measured using the MTT assay. The control group was set at 100%, and the data obtained in other groups were calculated as the percentage of control. ## indicates  *p* < 0.01 versus the control group; *∗∗* indicates  *p* < 0.01 versus the model group.

The cell viability in the OGD-R-treated group was 45.0 ± 2.4%, whereas those in the rLj-RGD3-treated groups (at 16, 8, and 4 *μ*mol/L) were 67.2 ± 2.7% (*p* < 0.01), 58.6 ± 2.3% (*p* < 0.01), and 55.4 ± 2.6% (*p* < 0.01), respectively, compared with the control group (set at 100%; Figure 3). The protective effect of rLj-RGD3 on the PC12 cells subjected to the OGD-R procedure was dose-dependent.

### 3.3. Effect of rLj-RGD3 on LDH Activity in the Supernatants

**Figure 4:**
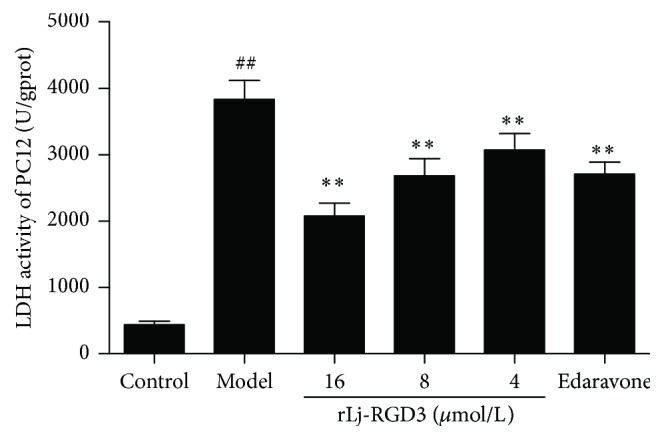
The effect of rLj-RGD3 on the LDH activity in the cell supernatants after OGD-R injury to the PC12 cells ($\bar{X}\pm SD$, *n* = 6). The LDH activity in the supernatant fluid was measured using a kit after PC12 cell injury was induced by OGD for 2 h and reperfusion for 24 h. ## indicates  *p* < 0.01 versus the control group; *∗∗* indicates  *p* < 0.01 versus the model group.

As shown in Figure 4, the model group was severely damaged compared with the control group, as indicated by a significant increase in the LDH activity (*p* < 0.01). In contrast, the LDH activity was substantially lower in the rLj-RGD3-treatment groups (*p* < 0.01), and the effect was dose-dependent. However, the LDH activity in the rLj-RGD3 high-dose group showed a less pronounced decrease (*p* < 0.05). The inhibition of LDH in the edaravone group was not significantly different from that in the rLj-RGD3 high-dose group.

### 3.4. Effect of rLj-RGD3 on GSH Content in the Cells

**Figure 5:**
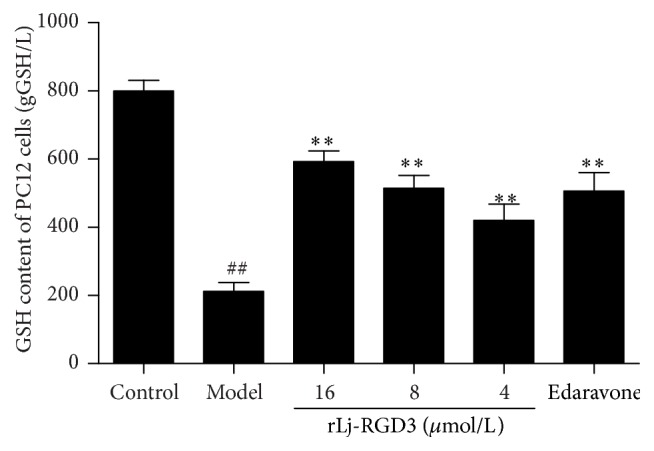
The effect of rLj-RGD3 on the GSH content in PC12 cells after OGD-R-induced injury ($\bar{X}\pm SD$, *n* = 6). The concentration of GSH in PC12 cells was measured using a kit after injury was induced by OGD for 2 h and reperfusion for 24 h. ## indicates  *p* < 0.01 versus thecontrol group; *∗∗* indicates  *p* < 0.01 versus the model group.

Our results (Figure 5) show that, compared with the control group, OGD-R caused a significant decrease in the GSH concentration that was dose-dependently attenuated by the rLj-RGD3 treatment. Significant differences were observed between the model group and the groups pretreated with rLj-RGD3. Furthermore, administration of the high dose of rLj-RGD3 significantly increased the content of GSH in the cells compared with those treated with edaravone.

### 3.5. Morphological Analysis of PC12 Cell Death

**Figure 6:**
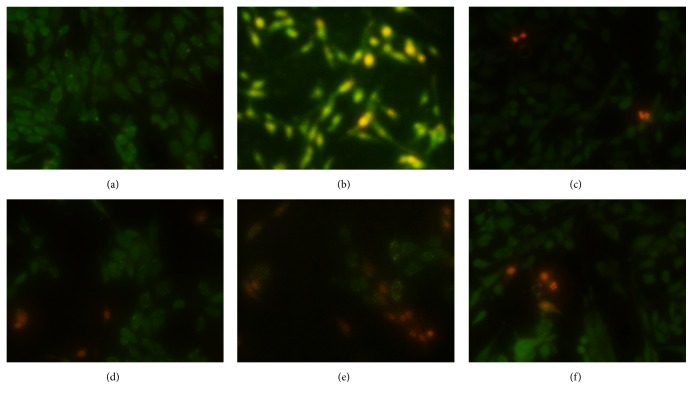
The effect of rLj-RGD3 on PC12 cell apoptosis after OGD-R injury as indicated by AO/EB fluorescence. (a), (b), (c), (d), (e), and (f) represented the control, model, rLj-RGD3 16, 8, and 4 *μ*mol/L dose groups, and edaravone control group, respectively. Most PC12 cells were apoptotic after OGD-R. In contrast, apoptosis of the PC12 cells was significantly reduced in the rLj-RGD3 high-dose group.

To study the morphological aspects of the protective effect of rLj-RGD3 on PC12 cell injury induced by OGD-R, we used AO/EB double-staining fluorescence analysis.  Figure 6 depicts the cell apoptosis in each group. The cells of the control group were round with green fluorescence. The most serious injury including orange staining, pyknotic nuclei, decreases in the numbers of adherent cells, and mostly elongated morphology was clearly observed in the model group. The majority of the cells of the rLj-RGD3 high-dose group exhibited the green fluorescence and the normal structure of living cells. Compared with the model group, apoptosis was significantly inhibited in the treatment groups. Moreover, improvement became more obvious with increasing doses of rLj-RGD3.

### 3.6. rLj-RGD3 Is Antiapoptotic in PC12 Cells

**Figure 7:**
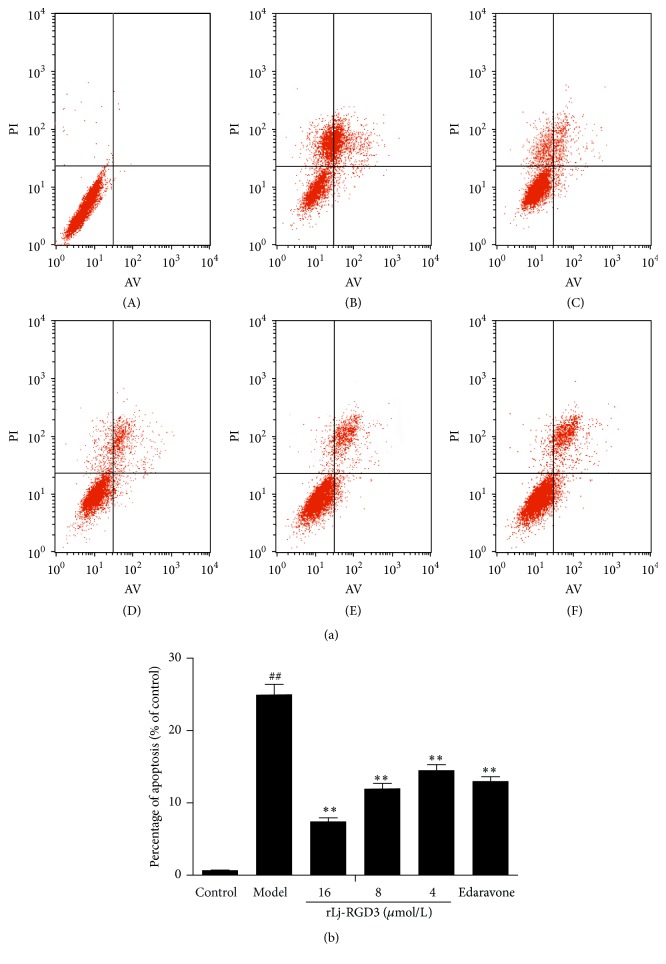
The effect of rLj-RGD3 on PC12 cell apoptosis after OGD-R injury as indicated by flow cytometry. In Figure 7(a), (A), (B), (C), (D), (E), and (F) represent the control, model, rLj-RGD3 16, 8, and 4 *μ*mol/L dose groups, and the edaravone control group, respectively. The bar graph showed the apoptosis rate in Figure 7(b). The numbers of apoptotic cells in the rLj-RGD3 groups were significantly lower than those of the model group. The results suggested that rLj-RGD3 had an antiapoptotic effect on PC12 cells after OGD-R. The data are shown as means ± SD, *n* = 6. ## indicates *p* < 0.01 versus the control group; *∗∗* indicates *p* < 0.01 versus the model group.

In the flow cytometric results, the upper left quadrant represents mechanical damage and necrotic cells; the lower left quadrant represents normal cells. The upper right quadrant represents early apoptotic cells and necrotic cells, and the lower right quadrant represents late apoptotic cells. We observed a large number of apoptotic cells in the model group.  Figure 7 shows a significant dose-dependent reduction in the early and late apoptotic cells in the rLj-RGD3 treatment groups compared with the model group, particularly for the rLj-RGD3 high-dose group. However, there was no significant difference in apoptosis between the rLj-RGD3-treated groups and the edaravone control group.

### 3.7. FAK and p-FAK Protein Expression

**Figure 8:**
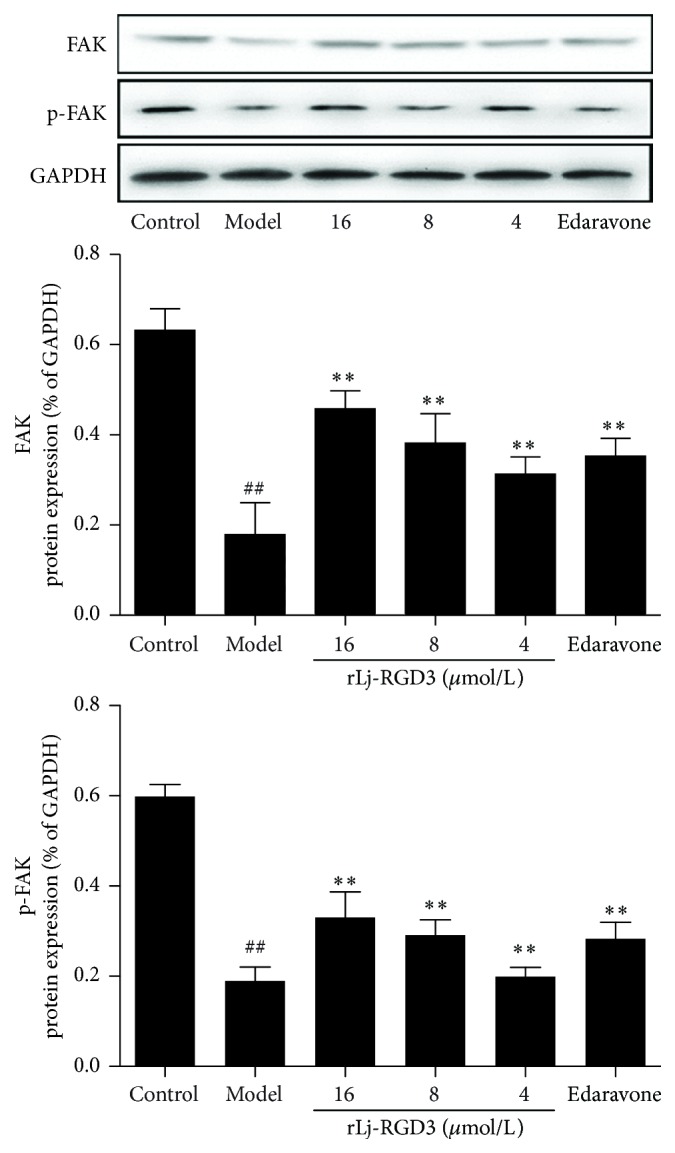
The effect of rLj-RGD3 on the expression of FAK and p-FAK proteins in PC12 cells after OGD-R injury ($\bar{X}\pm SD$, *n* = 3). After PC12 cell injury induced by OGD-R, we examined the levels of the proteins of interest, FAK, and its phosphorylated form, by western blotting. The protein bands shown in the figure were quantified after scanning and normalized to GAPDH. The results are presented as the mean ± SD of three independent experiments. ## indicates  *p* < 0.01 versus the control group; *∗∗* indicates  *p* < 0.01 versus the model group.

FAK is an important neuroprotective factor downstream from integrins. As shown in Figure 8, expression of both proteins was significantly lower in the model group than in the control group (*p* < 0.01). Treatment with rLj-RGD3 dose-dependently upregulated the expression of these proteins compared with the model group (*p* < 0.01). These results demonstrate that rLj-RGD3 can regulate FAK and p-FAK protein expression and protect the PC12 cells from the damage induced by OGD-R.

### 3.8. Expression of Caspase-3 and Bcl-2 Proteins

**Figure 9:**
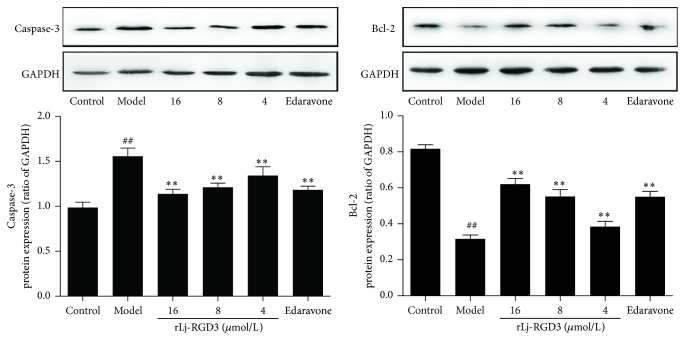
The effect of rLj-RGD3 on the expression of Caspase-3 and Bcl-2 proteins in PC12 cells after OGD-R injury ($\bar{X}\pm SD$, *n* = 3). The protein bands shown in the figure were quantified after scanning and normalized to GAPDH. The results are presented as the mean ± SD of three independent experiments. The results suggested that the studied drug inhibited the apoptosis of PC12 cell subjected to OGD-R. ## indicates  *p* < 0.01 versus the control group; *∗∗* indicates  *p* < 0.01 versus the model group.

Caspase-3 and Bcl-2 are important effectors in the regulation of cell survival or apoptosis. Their expression in the cell directly affects the cell state. The expression levels of both proteins were detected by western blotting. As shown in Figure 9, the expression of Caspase-3 and Bcl-2 proteins was significantly increased and inhibited in the model group, respectively. Compared with the model group, Caspase-3 expression was inhibited significantly, and Bcl-2 expression was increased in a dose-dependent manner in the rLj-RGD3-treated groups.

## 4. Discussion

Ischemic cerebrovascular disease is one of the most common causes of death worldwide [11]. Although an increasing number of studies have described the ischemic mechanisms, including the excessive production of free radicals [12, 13], altered calcium homeostasis [14], and N-methyl-D-aspartate excitotoxicity [15], accumulating evidence suggests that the cell death observed during the first few hours of cerebellar ischemia is the result of apoptosis as opposed to necrosis. Necrosis has been considered the predominant form of cerebellar damage generated by ischemia [16]. Moreover, ischemic damage to nerve cells disrupts a series of complex signalling pathways that affect the corresponding biological functions and thus brain function. The terminally differentiated profile of the brain is of particular relevance for cerebellar ischemia [17–19].

rLj-RGD3 is a recombinant protein with 3 RGD (Arg-Gly-Asp) motifs from the salivary gland secretions of* Lampetra japonica*, which is a histidine-rich and arginine-rich protein. The deduced amino acid sequence of Lj-RGD3 contains 118 amino acids including 17 histidine and 17 arginine. It is reported that histidine and arginine have cerebral ischemia-reperfusion and neuroprotective functions [20, 21], so we examined the related activities and mechanism in this paper. Our previous studies about rLj-RGD3 focused on the typical characteristics of RGD-toxin protein, such as platelet aggregation suppression, tumour metastasis, and angiogenesis inhibition [8]; the cerebral ischemia-reperfusion and neuroprotective functions of rLj-RGD3 were reported first in this paper. We examined whether rLj-RGD3 has protective effects on nerve cells through activation of pathways downstream of integrins due to its ability to bind to the specific site on integrins.

In vitro ischemic-like injury is typically induced by OGD in neurons [22]. Rat pheochromocytoma cells (PC12 cells) have become an important tool for in vitro studies of neurology [23, 24] because their morphological, physiological, and biochemical functions are similar to those of neurons and because they are easy to use for these types of experiments. PC12 cells are widely used in neurological research in pharmacology and physiology and in in vitro studies of ischemic injury [25]. In this study, we investigated the protective effects of rLj-RGD3 using a model of PC12 cell injury induced by OGD-R in vitro and have provided a preliminary discussion of its neuroprotective mechanism.

Lemons and collaborators have found that integrins are involved in the repair of nervous system damage [26]. This study was the basis for studies regarding the rLj-RGD3-mediated protection of nerve cells from injury through integrin receptors and provided theoretical support for the present study. Integrins can affect many complex downstream signalling pathways, including FAK, which is important in the downstream effectors of integrins in the nervous system. Studies have shown that increased levels of integrins on neuronal surfaces can enhance axonal growth. Furthermore, integrin receptors can affect nerve regeneration and functional recovery. Activation of FAK by integrins plays an important role in these processes. In addition, FAK activation can promote neuronal growth, and it has been suggested that FAK integrin signalling pathways play an important role in nerve regeneration [27, 28]. As indicated by the results of this study, compared with the control group, FAK and p-FAK protein expression were significantly reduced in the OGD-R group. However, rLj-RGD3 induced a dose-dependent increase in FAK and p-FAK protein expression levels, which suggested that rLj-RGD3 can improve the expression of the effectors FAK and promote its activation through activation of the integrin receptors. Therefore, rLj-RGD3 can play a key role in nerve repair and reduce the injury to PC12 cells resulting from OGD-R.

The Caspase family is an important regulator of apoptosis signal factors, and acute neuronal apoptosis is closely associated with the Caspase family. Caspase-3 is activated only during apoptosis [29]. The results showed that Caspase-3 protein expression was significantly increased in the model group, which is consistent with the discussion above. Additionally, rLj-RGD3 significantly reduced the expression of Caspase-3 in a dose-dependent manner. Our results suggested that rLj-RGD3 significantly inhibited Caspase-3 expression, thereby protecting PC12 cells from OGD-R-induced damage.

Bcl-2 is an important antiapoptotic factor downstream of the activation of the PI3K/Akt pathway. Many studies have demonstrated that it plays a key role in the inhibition of nerve cell apoptosis [30, 31]. The results show that Bcl-2 protein expression was significantly decreased in the model group, whereas rLj-RGD3 increased Bcl-2 protein expression levels. These results suggested that rLj-RGD3 enhanced antiapoptotic effects and could inhibit brain tissue damage by upregulating Bcl-2 protein expression.

## 5. Conclusions

In summary, this study demonstrated that rLj-RGD3 had obvious protective effects against OGD-R-induced neurotoxicity in PC12 cells. These effects appear to be mediated through integrin receptor-mediated activation of FAK and then through the activation of PI3K/Akt to increase Bcl-2 expression and downregulate Caspase-3 expression.

## Additional Points

*Highlights.* A RGD protein from* Lampetra japonica* (rLj-RGD3) blocked OGD-R-induced cytotoxicity. The inhibitory effect of rLj-RGD3 on cytotoxicity was associated with FAK. rLj-RGD3 suppressed the cellular apoptosis induced by OGD-R. Arginine and histidine-rich protein has neuroprotective functions. Our data suggest that rLj-RGD3 has a crucial role of neural protection.
